# Predicting gene expression changes from chromatin structure modification

**DOI:** 10.1038/s41540-025-00510-4

**Published:** 2025-04-15

**Authors:** Swayamshree Senapati, Inayat Ullah Irshad, Ajeet K. Sharma, Hemant Kumar

**Affiliations:** 1 School of Basic Sciences, Indian Institute of Technology Bhubaneswar, Argul, Odisha 752050 India; 2 Department of Physics, Indian Institute of Technology Jammu, Jammu, 181221 India; 3 Department of Biosciences and Bioengineering, Indian Institute of Technology Jammu, Jammu, 181221 India

**Keywords:** Computer modelling, Biological physics

## Abstract

Spatial organization of chromatin plays a critical role in gene transcription, but connecting population-averaged HiC data to functional outcomes remains a challenge. We present a computational framework linking HiC contact map to gene transcription. Utilizing a bead-spring polymer model informed by HiC contact maps, we generate an ensemble of 3D conformations for a given genomic locus. These conformations are then coupled to gene transcription levels through a Markov chain model, with transition rates derived from molecular dynamics simulations. The efficacy of this framework is demonstrated by simulating the perturbation of a CTCF-mediated TAD boundary, impacting the expression of *sox9* and *kcnj2*. Our model quantitatively reproduces experimentally observed changes in gene expression, revealing that the increased *kcnj2* transcription is a consequence of enhancers within the *sox9* TAD becoming accessible upon boundary disruption. Quantifying enhancer impact, our model can also identify functional enhancers. This framework enhances our understanding of the relationship between chromosome spatial architecture and gene regulation.

## Introduction

The regulation of gene expression is controlled by various elements, including cis-regulatory elements (CREs) such as enhancers and promoters, as well as trans-regulatory elements (TREs) like transcription factors (TFs) and coactivators^1–3^. Although genes and their regulatory elements may be scattered over long genomic distances; they physically interact in three-dimensional space to regulate gene expression. The specific contacts are achieved through genome organization, which brings together the regulatory elements associated with a target gene^4,5^. Any deviation from the optimal structure can alter the way regulatory elements interact, potentially leading to the misexpression of genes. Enhancers may disrupt their contact with natural promoters and exhibit aberrant interactions with alternative promoters, resulting in the dysregulation of gene expression. De Gobbi et al. have shown that a regulatory single-nucleotide polymorphism creates a pseudo-promoter-like element that interferes with the normal promoter-enhancer communication and causes aberrant gene expression of alpha-like globin genes in the primary human erythroblast cell line^6^. Oh et al. have also observed that the functional impairment of a promoter, along with its enhancer, can lead to the redirection of the enhancer and the formation of loops with nearby alternative promoters. This process was shown to activate disease-prone genes in a specific region (NUCKS1-RAB7L1) associated with Parkinson’s disease, as well as three other regions (CLPTM1L-TERT, ZCCHC7-PAX5, and PVT1-MYC) linked to cancer^7^. Moreover, multiple experimental studies have also shown that changes in any level of chromosome organization can lead to pathological outcomes. For example, intermingling of chromosomes 10 and 14 and translocation of chromosomes 7 and 10 lead to leukemia^8–11^. Mutation of lamin proteins, which help to anchor chromosomes to nuclear periphery, leads to disorders like lamina associated laminopathies^12^. Furthermore, the involvement of structural proteins such as CTCF and cohesin is crucial in facilitating the connection between enhancers and promoters, and thus the disruption of these proteins can lead to gene aberrations^13–16^. CTCF and cohesin loss has been shown to cause growth retardation and intellectual disabilities^17,18^. Nevertheless, quantitative understanding of the variability in gene expression levels attributed to genome organization remains largely unknown due to experimental limitations such as the limited resolution of HiC (High-throughput Chromosome Conformation Capture experiment), and the lack of multiple probes to simultaneously detect multiple genomic loci, and the phototoxicity of fluoroprobes in microscopy and in-situ fluorescence hybridization techniques^19,20^.

The physics-based chromatin models have served as important tools for understanding the intricacies of chromatin organization and contact between genetic elements within the nucleus. Drawing inspiration from principles of polymer physics and statistical mechanics, these models integrate experimental data on HiC and diverse omics data to simulate the dynamic spatial arrangement of chromatin segments within the nucleus^21–24^. Polymer based chromatin models have been used to give insights into different levels of chromosome organization, such as the formation of loops, topologically associating domains, A/B compartments, and intermingling of chromosome territories, and also to characterize one-dimensional genomic features in three-dimensional genome^23,25–28^. Giorgetti et al. employed a bead-spring model based on 5C data to forecast genetically verified crucial structural components within the TAD region of the Xic (X-inactivation center) in mouse embryonic stem cells. The polymer model could demonstrate asymmetric expression in the Xic as a result of changes in TADs^29^. Furthermore, Shukron and Holcman utilized a bead-spring polymer model based on a 5C contact map and incorporated long-range specific interactions. This allowed them to calculate the distribution of first encounter times and the conditional probability of three important genomic sites on chromosome X in female mice embryonic stem cells. Additionally, they were able to analyze live-cell imaging trajectories using simulations^30^. Genome organization has emerged as a critical factor in the complex regulation of gene expression, as evidenced by a growing body of research^31–33^. However, there remains a gap in the ability to directly infer the E-P kinetics from 3C-derived contact maps and 1D genomic data while preserving the statistical properties inherent in these experimental datasets and utilizing this information to infer the transcriptional activity of genes within those genomic loci. To address the need for a deeper understanding of the relationship between genome organization and gene expression, we are developing a computational framework that enables a direct and quantitative connection between the spatial arrangement of the genome and its regulatory impact on transcriptional activity.

To elucidate the relationship between gene expression and genomic organization, we developed a computational framework that utilizes the contact map derived from Chromosome Conformation Capture (3C) experiment or any 3C-derived experiment such as Chromosome Conformation Capture Carbon Copy (5C), High-throughput Chromosome Conformation Capture (HiC), and Capture HiC or cHiC (i.e., 3C library is prepared with targeted oligonucleotide capture and specific genomic contacts are enriched^34^). In principle, such models can also be used to explore how epigenetic marks, histone modifications, and chromatin remodelers collectively orchestrate the accessibility of transcriptional machinery to specific genomic loci, thereby modulating gene expression patterns.

In this study, we take a specific example of TAD boundary deletion to modify the genomic structure, and apply our computational framework to predict the gene expression level corresponding to this change. Within our computational framework, first we construct a polymer model capable of providing us with a three-dimensional configuration of the chromatin with a cHiC contant map as input. The polymer model allows us to infer binding and unbinding rates and capture transient enhancer-promoter interactions, which are not directly accessible from 3C derived contact maps, while preserving its statistical properties. Utilizing this polymer model, we were able to discern alterations in enhancer-promoter (E-P) interactions following the deletion of TAD boundaries. Then, by combining the simulated trajectory with a kinetic model of gene expression, we accurately quantified the variations in gene expression resulting from modifications in chromatin structure. Notably, our analysis established the specificity of enhancers in regulating gene expression and revealed the importance of chromatin organization in modulating specific E-P interactions in governing gene expression. We also demonstrate that the specific E-P interaction leads to dissimilar changes in the expression levels of two genes situated within adjacent TADs. Our framework emerges as a means to establish a quantitative link between chromatin structure and the expression of encoded genes. By capturing the non-linear relationships inherent in the complex regulatory network, our modeling framework will enhance our fundamental understanding of the impact of genome architecture on gene regulation.

## Results

### Polymer modeling reveals spatial conformation of chromatin and physical characteristic of TAD arrangement

**Fig. 1:**
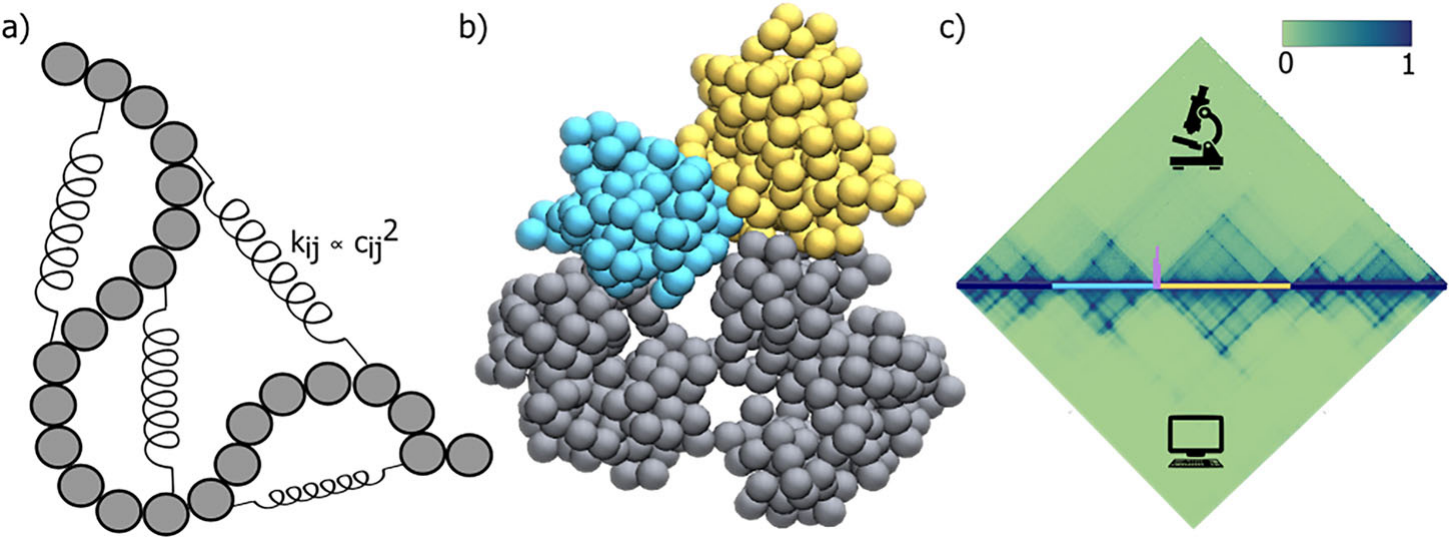
Bead spring polymer model of wildtype sox9-kcnj2 loci. **a** Schematic representation of polymer model of chromatin is represented by chain of beads where each bead represents 10 kb genomic region and the spring represents the spring constant between two beads and the spring constant (k_ij_) between two beads i and j is proportional to square of the contact probability (c_ij_) between i and j; **b** Time snapshot of the 3D structure of WT cell (cyan and yellow globular domains show the kcnj2 and sox9 TADs, respectively, and silver beads show other genomic regions of the considered 6 Mb region (drawn using VMD^84^); **c** The contact matrices derived from cHiC experiments performed in E12.5 limb buds (top) and the polymer model (bottom) for WT cell (Pearson correlation = 0.969). Schematic of the genomic region highlights kcnj2 TAD (cyan line) and sox9 TAD (yellow line) separated by CTCF boundary (purple marker).

To generate three-dimensional (3D) conformations of a given segment of chromatin, we generate a series of polymer confirmations from the MD simulation trajectory (Fig. 1a). We repeat the MD simulations with 200 different initial conditions to capture different possible conformations and minimize initial condition bias. In total, 4 ×10^6^ distinct configurations were recorded to represent the ensemble of 3D configurations of the chromatin segment under investigation. To assess the quality of the simulated 3D structure with the actual chromatin organization within the nucleus, we calculated the Pearson correlation coefficient between the model-derived contact map and the experimental cHiC contact map. We obtain the Pearson correlation coefficient of 0.96, which indicates a strong correlation (Fig. 1c) between the simulated 3D structure and the experimentally measured chromatin structure. Such polymer models have demonstrated their efficacy in elucidating the spatial conformation of chromatin from cHiC contact maps^21,23,24,30,35,36^.

The generated ensemble of 3D chromatin structure was systematically analyzed to identify distinctive characteristics of the three-dimensional organization of the genomic loci. Our observations revealed the formation of structures resembling globules for each kcnj2 and sox9 TAD. The beads corresponding to a TAD form a single globule and form dynamical connections with other beads in the globule (Fig. 1b). Furthermore, beads representing the kcnj2 and sox9 TADs exhibited a striking non-mixing behavior relative to the mixing of intra-TAD beads, such that TADs maintain their respective independent spaces within the 3D conformation.

To correlate gene regulation and chromatin structure, spatial interactions of encoded regions with regulatory elements were investigated. In particular, an exhaustive list of all enhancers encoded in this chromatin segment and associated with sox9 and kcnj2 was created^37,38^. Subsequently, a focused analysis of enhancers and promoter interactions from the simulated ensemble of configuration was carried out. Our investigations revealed that all 44 enhancers associated with sox9 as identified experimentally by Despang et al. were situated within the confines of sox9 TAD. In contrast, the kcnj2 locus resided in an adjacent TAD, spatially separated from all the enhancers. This elucidates the role of TAD boundaries as insulating elements that restrict the interactions between enhancers and promoters located in neighboring TADs^39–41^.

### MD simulation trajectories quantify three-dimensional E-P interactions and highlight the role key-enhancer in gene transcription

Various experimental studies using techniques including 3C, 4C, 5C, and ChIA-PET, have demonstrated that all active enhancers form physical contacts with promoters via chromatin looping or some form of tracking mechanism, despite genomic separation from the promoter^42^. Given that our modeling approach captures the statistical fluctuations of chromatin structure around the HiC data through temporal evaluation of configuration, we can quantify interactions of enhancers and promoters in 3D space and infer the kinetics of these contacts.

**Fig. 2:**
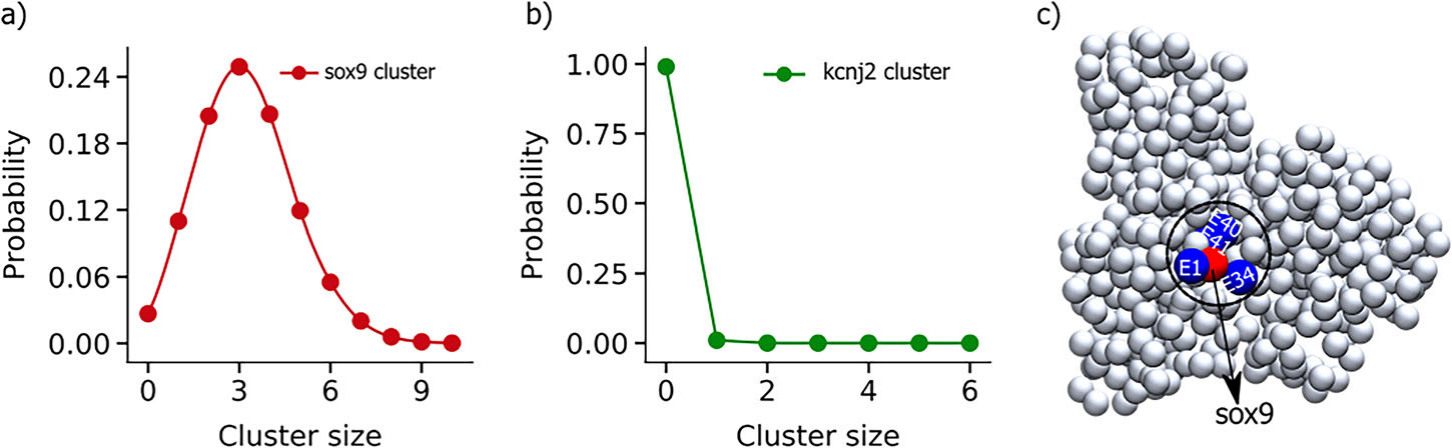
Enhancer-promoter interaction in wildtype sox9-kcnj2 loci. The probability distribution for different sizes of enhancer clusters computed from MD trajectories around **a** sox9 and **b** the kcnj2 promoter in the WT cell; **c** The polymer snapshot (drawn using VMD^84^) shows the enhancers E1, E34, E40, and E41 (blue beads) around the sox9 promoter (red bead).

To quantitatively investigate the nature of E-P contacts inside the nucleus, we selectively look into the pairwise contacts between 44 enhancers and promoters corresponding to both genes, namely, sox9 and kcnj2. These enhancers were located at distances ranging from tens to hundreds of kilobase pairs from the promoters. Our analysis reveals that enhancer beads form a dynamic cluster around the promoter bead, with enhancer beads continuously moving in and out of contact range (1.2*σ*) of the promoter bead. We track the total number of enhancers surrounding each promoter at a given time and its variation with time. The average number of enhancers surrounding the sox9 is 3.21, with a median value of three (Fig. 2a). On the other hand, the kcnj2 promoter in the neighboring TAD is in contact with only one enhancer with a miniscule contact probability of 0.01 (Fig. 2b). A closer look reveals that among 44 enhancers, only four enhancers namely E1 (chr11 : 111,519,300 - 111,520,200), E34 (chr11 : 112,671,913 - 112,672,982), E40 (chr11 : 112,982,498 - 112,983,853) and E41 (chr11 : 113,008,929 - 113,009,857) have significant contacts with the sox9 promoter, and other enhancers form relatively intermittent contacts with the promoter (Fig. 2c). However, the number of enhancers coming in close contact with the sox9 promoter fluctuates and occasionally can reach up to 10 (Fig. 2a). The probability of a specific number of enhancers being in contact with promoters is shown in Fig. 2a.

To identify the most effective enhancers out of the 44 enhancers associated with sox9, we conducted an analysis of the binding affinities between individual enhancers and the sox9 promoter. Among the 44 enhancers examined, E41 has the highest affinity for the sox9 promoter as it spends 26.87% of time in contact with the sox9 promoter, out of which it spends 2.9% of time as a single with the promoter. It spends 18.7% of its time as a diad and 50.1% of its time as a triad (Table I). Similarly, E1, E40, and E34 exhibit 24.98%, 18.55%, and 16.5% time in contact with promoters, respectively. E1 spends 4.4% of its time as a single, 20.8% of its time as a diad, and 42% of its time as a triad out of all the time it attaches to the promoter. Moreover, our analysis also suggests that E34 and E40 spend 4.1% and 4.38% time as singles, 22.5% and 22.2% time as diads and 34.3% and 49.8% time as triads, respectively, out of the time they attach themselves to the sox9 promoter. Other enhancers have a smaller chance to be in contact with the sox9 promoter (0.15–12.74%). Based on this analysis, we propose that these four enhancers are most important to effectively regulating the sox9 gene expression. The findings of this study indicate that the effective enhancers exhibit cooperative regulation with other enhancers for gene regulation.

**Table. I:** Cooperative nature of enhancers. Probability of the enhancers to be in simultaneous contact with other enhancers

Enhancer	Location (chr11, in bp)	Single	Diad	Triad
E1	111,519,300 - 111,520,200	4.4%	20.8%	42.1%
E34	112,671,913 - 112,672,982	4.1%	22.5%	34.3%
E40	112,982,498 - 112,983,853	4.3%	22.2%	49.8%
E41	113,008,929 - 113,009,857	2.9%	18.7%	50.1%

### CTCF TAD boundary deletion leads to TAD fusion, resulting in significant alterations to enhancer-promoter interactions and the spatial organization of enhancer elements

**Fig. 3:**
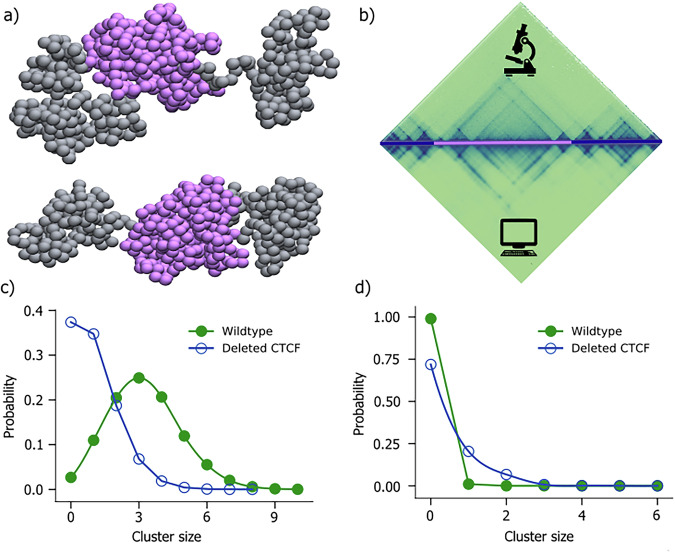
Chromatin configuration of DELC sox9-kcnj2 loci. **a** A representative 3D configuration of DELC chromatin segment cell (Top and bottom structures represents front and back of the 3D ensemble) (drawn using VMD^84^); **b** The contact matrices derived from cHiC experiments performed in E11.5 limb buds (top) and the polymer model (bottom) for DELC cell (Pearson correlation = 0.92). Purple line in the genomic region highlights merged TAD shown in (**a**)); **c**, **d** The probability distribution curve of enhancer clusters of different sizes around the promoters shows the enhancer clustered around the sox9 and kcnj2 promoters, respectively. The green curve shows the probability distribution plot for the WT and the blue curve for the DELC cell.

Disruption of chromatin organization can have significant consequences on gene regulation, as it can result in the occurrence of ectopic interactions between enhancers and their target promoters^41^. Despang et al. have shown that deletion of all four CTCF binding domains present at the boundary of sox9 and kcnj2 TADs in chromosome 11 of E12.5 mouse limb bud cells leads to the merging of both TADs. Expression analysis reveals that the deletion of major CTCF binding domains leads to a two-fold increase in the kcnj2 expression level, whereas the sox9 expression level decreased by 20% compared to WT cell. Our goal is to reproduce this expression change through our computational framework and then understand how the modulations in E-P interactions give rise to such changes in gene expression. The analysis of molecular dynamics trajectories of chromatin provides a valuable tool for investigating alterations in the dynamics of regulatory elements arising from such structural disruptions. To this end, we simulate the CTCF boundary deletion region between sox9-kcnj2 TADs. Using the same methodology employed for the WT cell, we constructed an ensemble of 3D conformation of the DELC cell (Fig. 3a). The contact matrix generated from this ensemble of configurations is highly correlated with the cHiC contact map with Pearson correlation coefficient of R = 0.92 (Fig. 3b). The examination of the 3D conformation reveals that in the DELC cell, the two distinct globules representing individual TADs encompassing the sox9 and kcnj2 genes in the WT cell, are merged into a single globule (Fig. 3a).

The merging of TADs also impacts the E-P interaction. Based on the ensemble of 3D structures generated using the MD simulation from cHiC data, we compared the E-P interaction for both cases (WT and DELC). To quantify the changes in E-P interactions resulting from the TAD fusion, we compared enhancer cluster size surrounding both the genes in WT and DELC cells. The average number of enhancers forming clusters around the sox9 was 3.21 before deletion while after deletion this number reduces to 1.04, signifying the reduced accessibility of enhancers post CTCF boundary deletion. On the other hand, the average number of enhancers for kcnj2 increases from 0.001 to 0.36 indicating increased contact with enhancers upon TAD fusion.

To get more insight into the E-P interaction, we compared the distribution of the size of the enhancer cluster before and after CTCF boundary deletion for both the genes. Enhancer cluster size distribution of sox9 shows that E-P interaction significantly decreased for the sox9 gene after TAD boundary deletion (Fig. 3d), as the peak of the probability distribution curve corresponding to the size of enhancer-cluster around sox9 promoter shifts from 3 to 0 (Fig. 3c). On the other hand, the probability peak remains at 0, but the magnitude of the probability increases from 0.01 to 0.203 in the DELC cell (Fig. 3d).

**Fig. 4:**
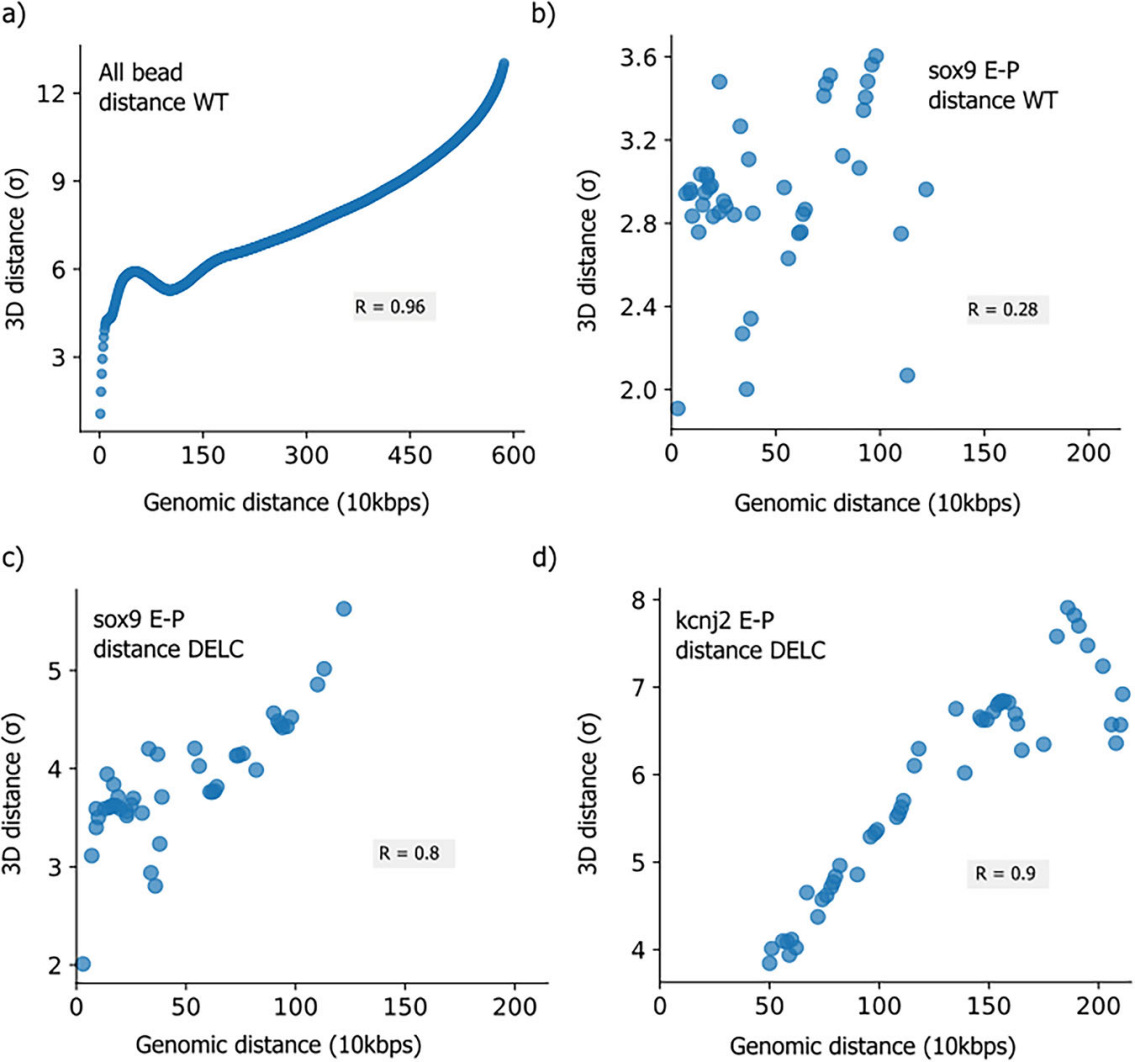
The genomic distance vs. 3D distance plot. The genomic distance (in 10 kbps or one monomer) vs. 3D distance plot (in *σ* units) for **a** all beads in WT cell; **b** E-P distance for sox9 gene in WT cell; **c** E-P distance for sox9 gene in DELC cell; and **d** E-P distance for kcnj2 gene in DELC cells, respectively. Here, R represents the Pearson correlation coefficient.

Next, we ask the question: are genomic distance from promoters and average physical distance with promoters correlated? To answer this, we calculate the average spatial distance for different genomic distances. We observed a significant correlation (R = 0.96) between the genomic distance and average physical distances between different chromatin regions and sox9 promoters within a TAD (Fig. 4a). This correlation is similar to normal polymer behavior, which follows a scaling law between monomer separation and spatial separation^25,40^. This behavior imposes constraints on genomic regions, effectively preventing the interaction between the distal loci within a TAD. However, interestingly, our analysis reveals that enhancers do not show any correlation between their genomic distance and physical 3D distance from their promoters (Fig. 4b). These results highlight that the positions of enhancers within the genome are highly adaptable and context-sensitive in their role of controlling gene expression, ensuring that genes can be activated even if they are far apart on the DNA strand.

Contrary to our findings in WT TADs, in DELC, we observed a strong positive correlation (R _(sox9)_ = 0.80, R_(kcnj2)_ = 0.90) between the genomic and physical distances of the enhancer and the promoter for both the genes, sox9 and kcnj2 (Fig. 4c, d). This implies that after TAD boundary deletion, enhancers lose specific arrangement inside the TAD, and exhibit the relationship similar to the other non-specific genomic regions. This revelation highlights the profound impact of TAD boundaries on orchestrating E-P interactions.

In addition to these observations, we find that enhancers are affected differently depending upon genomic distances from the TAD boundary, despite their co-localization within the same TAD. Notably, the average spatial distances between the enhancers and the sox9 promoter, located near the TAD boundary (e.g., mm627 at chr11 : 112,671,913 - 112,672,982, E1 at chr11 : 111,519,300 - 111,520,200, E2 chr11 : 111,546,750 - 111,548,800, hs1467 chr11 : 111,689,219 - 111,690,538), were found to be approximately doubled in the DELC cell compared to the WT cell. On the other hand, enhancers located far away from the boundary exhibit relatively small (1–26%) changes to the average spatial distance with sox9 promoter. This intriguing finding implies that the 3D chromatin conformation, in conjunction with genomic position, plays a pivotal role in governing E-P interactions and, consequently, in gene regulation.

### Parameterization and validation of E-P kinetics

**Fig. 5:**
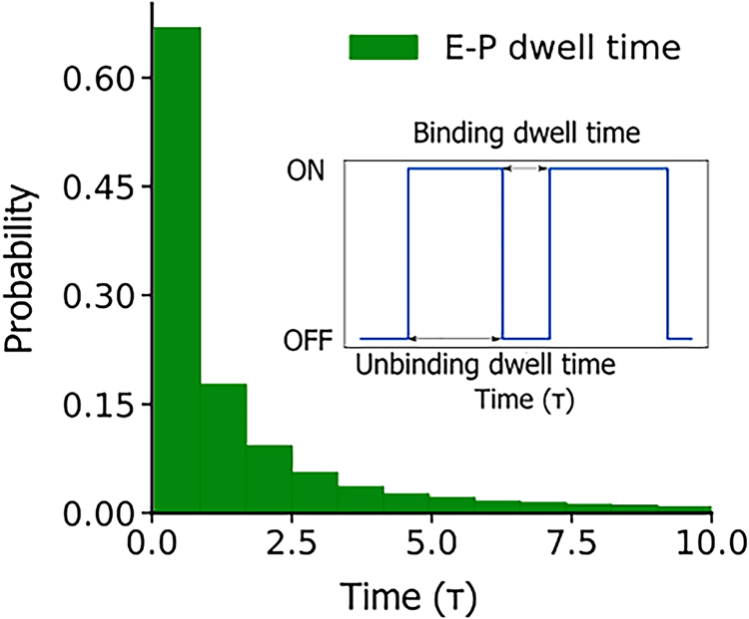
Dwell time distribution of enhancer (E1) bead with sox9 on promoter bead pair. The histogram plot of sox9 promoter and E1 enhancer shows an exponential distribution. The inset shows the schematic of binding and unbinding of one enhancer with its target promoter. The time is in τ (reduced) units.

The observed changes in E-P contacts are likely to be responsible for the transcription changes observed in the experiment. However, quantitative correlation and predicting changes in transcription levels due to E-P interaction modifications remain challenging. As gene expression is reliant upon the E-P interactions, we quantify the binding and unbinding of different enhancers with a given promoter from the MD simulation trajectories. In particular, we calculated the binding and unbinding rates of all identified enhancers with both the promoters, as these rates influence the transcription level of the target promoter^43^. The rates were calculated by fitting an exponential function to the dwell time distribution in each of the states of a given E-P pair which were computed from MD simulation trajectories (Fig. 5). (See Supplementary Section S5 and Supplementary Fig. S5).

**Fig. 6:**
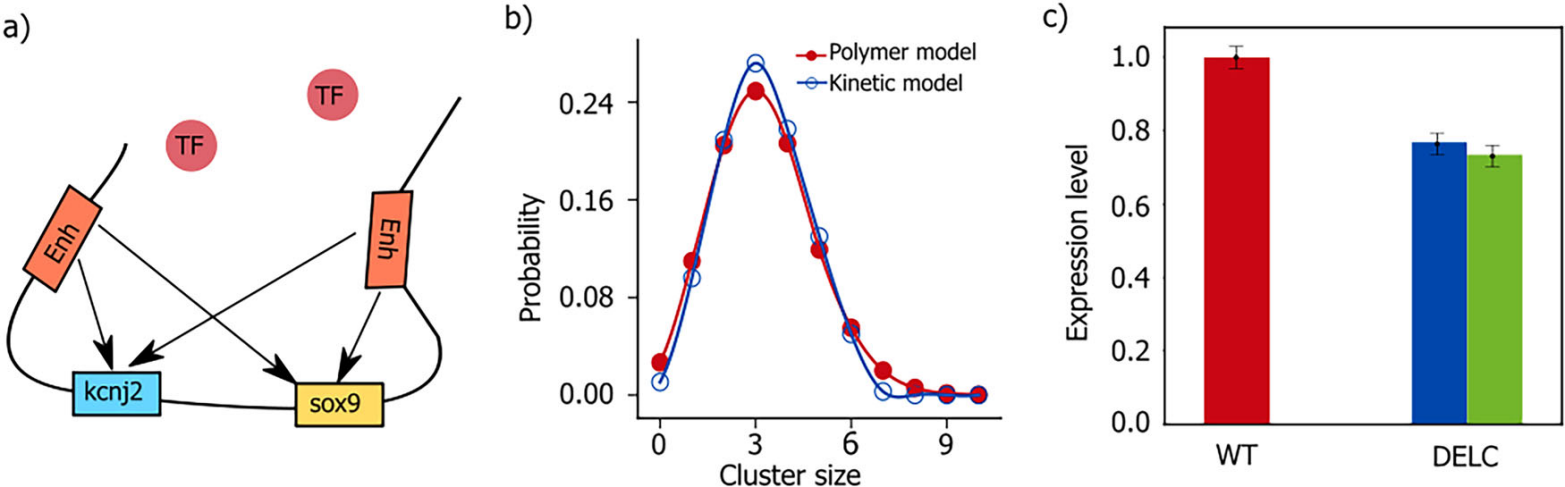
Kinetic model predicts transcriptional change in sox9 gene due to TAD boundary deletion. **a** Schematic of the kinetic model: kcnj2 and sox9 promoters can bind to enhancers with their respective binding and unbinding rates on the genomic location. The transcription factors are represented by spheres which move freely in the nucleoplasm and can bind/unbind with both the promoters; **b** Validation of the kinetic model. The probability distribution plot of enhancer cluster size around the sox9 promoter in polymer simulation (red curve) and kinetic model (blue curve) is shown; **c** Relative expression of sox9 in E13.5 limb buds for WT and DELC cells. Bars represent the mean of condensate size and error bars represent the standard deviation. The WT gene expression is normalized to one both for experiment and simulation. For the DELC cell, the blue bar shows the experimental result and the green bar shows the simulation result.

We observed that binding/unbinding rates exhibit dependence on the presence of enhancers already paired with promoter. To quantify this, we compute the binding and unbinding rates for a given enhancer for different E-P cluster sizes. We observed a linear decrease (increase) in the binding (unbinding) rates with increasing cluster size (Supplementary Fig. S6). This indicates that it becomes progressively difficult for enhancers to bind a promoter site as E-P cluster size increases. Thus, the number of enhancers bound to a promoter cannot go beyond a threshold number, determined by the thermodynamics of E-P binding. To further understand the dynamics of E-P interactions responsible for the expression of sox9 and kcnj2 genes, we developed a stochastic kinetic model of gene expression. The model specifically accounts for the binding and unbinding of each enhancer with the promoters of both genes (see Fig. 6a for details). We simulate the model using Gillespie’s algorithm. The input rate parameters for the simulations are computed using MD simulation trajectories (see Supplementary Section S5). Using our kinetic model, first we validate if it (Fig. 6a) faithfully captures the E-P interaction simulated through our constrained polymer model. To this end, we compute the average E-P cluster size from 100 independent simulation trajectories produced by our kinetic model and compare the distribution of E-P cluster size from both simulations (Fig. 6b). As shown in the Fig. 6b, cluster size distribution of the WT sox9 gene matches quite well with the polymer simulation model. This shows that our kinetic model can accurately capture the E-P dynamics.

### Markov chain integrating E-P interaction and TF binding predicts the transcription changes in the structural variants

While E-P binding plays a crucial role in transcription regulation, TFs also participate in the process. Xiao and co-workers developed a kinetic model based on the E-P and TF-promoter binding rates to predict the transcription level of a gene. We employ a similar approach to predict the level of transcription for a given set of E-P binding/unbinding rates. In this analysis, E-P binding and unbinding rates were computed using the simulation trajectories, whereas TF binding and unbinding rates were chosen to be the same as provided in the kinetic model of Xiao and coworkers^44^.

We incorporate the influence of TF as an independent binding/unbinding event (see Method Section “Kinetic model”). In the model, we assumed that the expression level of a gene is directly proportional to the average number of enhancers and weighted number of TFs (cluster) attached to the promoters^45^. We use the same weight factor for both WT and DELC cells. Remarkably, we found that the relative decrease in gene expression levels of sox9 measured in the WT cell compared to the DELC cell matches the findings of the experiments by Despang et al. aforementioned study (Fig. 6c). Our model was able to quantitatively predict the gene expression changes due to TAD boundary deletion. We could directly connect the structural changes with the gene regulation through this approach. Our approach not only gives a simple tool for predicting gene expression but also provides insights into the impact of E-P interaction on gene regulation.

### Newfound enhancer-accessibility drives large change in gene expression level of kcnj2 gene post-boundary deletion

**Fig. 7:**
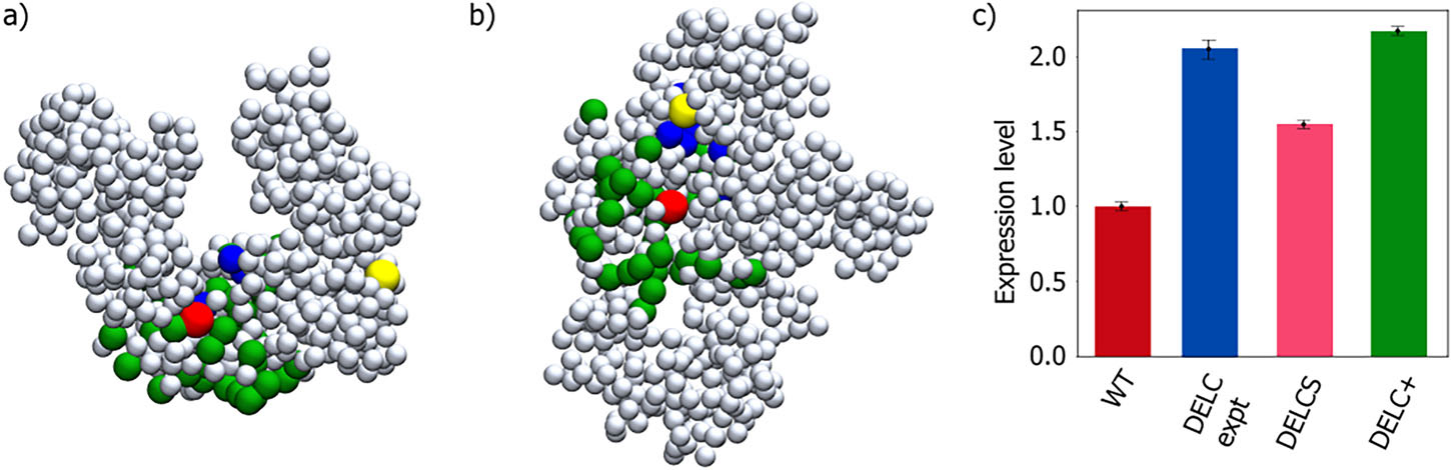
3D chromatin configuration highlighting enhancer-promoter specificity and kcnj2 expression changes in WT and DELC Cells. 3D configuration of chromatin segment captured from MD trajectories highlighting the representative positions of enhancers with sox9 and kcnj2 promoters in **a** WT cell and **b** DELC cell. Sox9 promoter, kcnj2 promoter, specific and non-specific enhancers are represented by red, yellow, blue and green beads, respectively, and other beads are made light gray (drawn using VMD^84^); **c** Relative expression of kcnj2 in E13.5 limb buds for WT and DELC cells. DELCS and DELC+ bars show the relative expression of kcnj2 considering only the specific enhancers and considering both specific and non-specific enhancers, respectively, and error bars represent the standard deviation over 100 initial conditions. The WT gene expression is normalized to one both for experiment and simulation.

To understand the remarkable increase in the kcnj2 gene expression levels in the DELC cell with respect to the WT cell, we closely examine contacts of the kcnj2 promoter with various enhancers reported in different studies^38^. We identified a total of nine possible enhancers specific to kcnj2. Out of these nine, six enhancers (mm628, mm629, mm630, mm631, mm632, and mm2181) affect only kcnj2, and the three enhancers (mm627, mm634, and mm1285) shared by both sox9 and kcnj2. We refer to these nine enhancers as kcnj2-specific enhancers. Through MD trajectory, we observed that all kcnj2-specific enhancers, which were identified from the Vista enhancer browser, were present in the sox9 TAD in the WT cell^38^ (Fig. 7a) and rarely made contact with the kcnj2 promoter. However, they form a cluster around kcnj2 and frequently come in contact with the promoter after TAD boundary deletion (Fig. 7b). This suggests that these enhancers may not be active in WT of this particular cell type and might have been encoded to be useful in alternate chromatin configuration corresponding to other cells. Our findings also suggest that structural elements such as the TAD boundary can be instrumental in enhancers selectivity^46^.

Next, we examine if the E-P interaction with the kcnj2-specific enhancers is responsible for enhanced transcription of kcnj2 in DELC. In other words, the evidence of E-P contact in spatial arrangement of chromatin can be attributed to the functionality of an enhancer. We propose to use the spatial contact information about E-P interactions easily obtained from the simulated 3D genome structure, as a computational tool for identifying active enhancers in a given chromatin conformation. To quantify the impact of these specific enhancers, we computed the relative contributions of the specific and nonspecific enhancers in the expression (Fig. 7c). Our results show that specific enhancers contribute to nearly 70% of the total gene expression in the DELC cell (Fig. 7c), thereby, the significant increase in the kcnj2 expression can be attributed to pairing with specific enhancers. Based on this, we believe that physical distances between enhancer and promoters, extracted from MD simulations, can be used to identify the active enhancers.

### The model predicts up to an 8-fold misexpression of the *Pax3* gene due to EP boundary deletion in the E11.5 mESC cell line

**Fig. 8:**
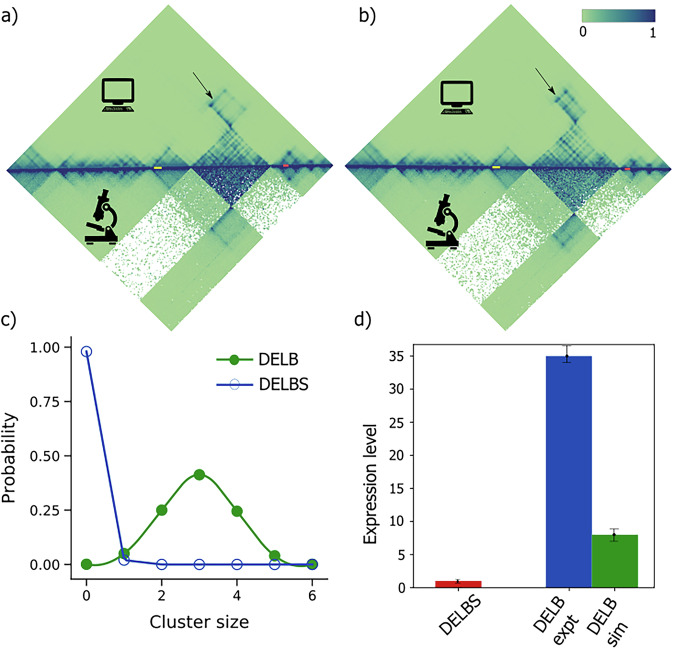
Model predicts misexpression of pax3 gene due to E-P boundary deletion. **a** The contact matrices derived from cHiC experiments performed in E11.5 limb buds (bottom) and the polymer model (top) for DELBS of pax3 genomic loci from 73 Mb to 79 Mb of chromosome 1 in mESC cell line (Pearson correlation = 0.95); **b** The contact map for DELB mutant from cHiC experiment (bottom) and polymer simulation (top) for the same genomic loci (Pearson correlation = 0.96). The yellow and red lines show the enhancers and pax3 gene regions in the genomic loci and the arrow represents the interaction change from DELBS to DELB mutants; **c** The probability distribution of enhancer cluster size around pax3 promoter in DELBS (blue curve) and DELB (green curve) predicted from polymer simulation; **d** The bar-plot shows the gene expression changes from DELBS to DELB. The red bar shows the gene expression level for DELBS, normalized to one for both simulations and experiment. The blue bar represents the gene expression change determined from experiment and the green bar shows the expression level for pax3 in DELB mutant predicted from the kinetic model. The error bars represent the standard deviation over 100 initial conditions for the simulation prediction.

To examine the versatility of our approach, we applied our computational framework to a 6 Mb genomic region encompassing the *Pax3* gene locus on chromosome 1 in the E11.5 mESC cell line. Chromatin interaction data (cHiC) for the chromosome 1 region spanning 73 Mb to 79 Mb, at a resolution of 10 kb, were obtained from publicly available datasets (GEO accession number: GSE92291)^47^. These data correspond to two mutant cell lines: DELB, characterized by a large deletion that includes the enhancer-promoter (E-P) boundary between *Epha4* and *Pax3*, and DELBS, where a similar deletion occurs without disrupting the E-P boundary^47,48^. Using a polymer model with 600 beads, we reconstructed the three-dimensional chromatin structures for both DELB and DELBS mutants. We calculated the Pearson correlation coefficients between the simulated and experimental contact maps for the *Pax3* locus. The correlation was 0.95 for the DELBS mutant (Fig. 8a) and 0.96 for the DELB mutant (Fig. 8b), indicating a high correlation between the model and experimental data.

Next, we analyzed the enhancer cluster size for the *Pax3* promoter, located at 78.09 Mb (mm9 genome assembly). The relevant enhancers in the E11.5 mESC cell line—hs1507, mm1036, mm1042, mm1044, mm1046, and hs1635—are located within the 75.7–75.8 Mb region (mm9 genome assembly)^37–39^. Cluster size distributions were determined for both mutants, revealing that the average cluster size in DELBS was 0.02, while in DELB, it was 2.97 (Fig. 8c). These values are closely aligned with experimental data, supporting the conclusion that deletion of the E-P boundary can result in ectopic interactions between *Pax3* and its distal enhancers^39,47,48^.

We further estimated the binding and unbinding rates of the six enhancers to the *Pax3* promoter and used the kinetic model to quantify the expression levels of *Pax3* in both mutants. The model predicted an ~8-fold increase in *Pax3* misexpression in the DELB mutant relative to DELBS (Fig. 8d). However, experimental observations indicate a 35-fold misexpression of *Pax3* in DELB compared to DELBS^48^. This discrepancy suggests that, while our model is good for qualitative prediction of gene misexpression (capturing up to 10-fold changes), further refinements are necessary to accurately predict the quantitative match with the experimental data induced by large-scale genomic alterations. Additionally, the model can serve as a tool for generating hypotheses to identify previously undiscovered enhancers within the regulatory region.

## Discussion

Our study introduces a computationally efficient approach to predict gene expression based on the spatial organization of chromosomes derived from any chromosome conformation capture experiment contact map. By conducting a systematic analysis of 3D chromatin organization, we unveil specific folding patterns within the chromosome conformation (Figs. 1c and 3b). Our polymer model was able to show the changes of E-P interaction due to genome rearrangements like TAD boundary deletion (Fig. 3c, d). Our model also gives insights into the role of chromatin conformations into determining the specificity of E-P interaction (Figs. 7 and 8). The temporal evolution of E-P contacts extracted from trajectory was shown to be a good indicator of the functionality of an enhancer. We find interesting aspects of E-P interaction using polymer simulation trajectories. We observe that there is very low correlation between the physical distance and genomic distance for sox9 promoter and enhancers as shown in Fig. 4b. The lower correlation between sequence distance and physical distance can be attributed to the spatial conformation of chromatin, a feature supported by experimental findings such as those reported in references^49–51^. This indicates how regions that are sequentially distant along the chromatin can be brought into close spatial proximity by organizational features.

We also observe that the binding rate decreases and the unbinding rate increases as cluster size grows (Supplementary Fig. S6). This phenomenon may result from volume exclusion effects and molecular crowding^52^. The fractal nature of the polymer likely contributes to this behavior by restricting enhancer binding as cluster size increases, reducing the effective volume available for molecular interactions due to crowding at length scales below hundreds of nanometers^53,54^.

Several modeling studies have yielded valuable insights into the fundamental aspects of chromatin organization. However, these studies do not make quantitative predictions of gene expression for different chromatin conformations and gene regulation^21,23,35,36^. In contrast, our framework offers a highly effective approach to quantify relative gene expression for different chromatin conformations. This quantification helps to develop an understanding of the intricate relationship between chromatin conformation and the regulation of gene expression. By taking a specific case of structural disruption through TAD boundary deletion, and predicting the transcription changes consistent with experimental results, we validate the ability of our model to make quantitative prediction of gene expressions. Our study introduces a simple framework that captures the key features of structure-dependent gene regulation, such as alteration of E-P interactions resulting from significant CTCF deletions across TAD boundaries, and accessibility of the promoters for TF binding. The present analysis underscores the significance of the spatial genomic architecture in governing the interaction between enhancers and promoters^55–58^.

Our finding also uncovers the impact of specific chromatin folding on establishing the enhancer specificity. We conducted a thorough search across many databases to identify all the E-P interactions of the genes sox9 and kcnj2 within the specified loci for E11.5 limb bud cells in mouse embryonic stem cells^26,37,38,59,60^. Specifically, we demonstrate that a few enhancers specific to kcnj2 are inaccessible to promoter sites due to presence of the TAD boundary, and are able to make contacts after the deletion of TAD boundaries. Among the nine enhancers identified computationally, only three enhancers, namely mm627, mm628 and mm634, have been experimentally validated as kcnj2 enhancers in these cells^38^. Our analysis confirms that only these enhancers are capable of forming contacts with kcnj2 promoter, but are restricted by the specific genome folding achieved with the help of TAD boundaries. However, deletion of the TAD boundary removes these topological constraints, and E-P contacts are possible (Fig. 7a, b). Our model demonstrates significant potential in predicting chromatin structural changes and their impact on gene expression, as evidenced by its ability to accurately replicate contact maps and good for qualitative predictions of gene misexpression levels of sox9, kcnj2 and pax3 gene in mESC cell line (Figs. 6c, 7c and 8d). However, its limitation lies in underestimating extreme expression changes, such as the observed 35-fold misexpression of Pax3^48^. This discrepancy underscores the need for further model refinements, including the integration of additional regulatory mechanisms or more intricate chromatin dynamics, to achieve a precise quantitative alignment with experimental data resulting from large-scale genomic alterations. Since our model takes into account all the enhancers, the difference between the predicted and actual gene expression can be used as a mean to generate experimental studies to uncover previously unidentified enhancers within the regulatory region.

Although we have used our model to predict the change in transcription due to deletion of the TAD boundary in limb bud cells, it can be easily extended to other cell types and means of chromatin structural changes. Our model can also be applied to study different aspects of TAD architecture, like TAD size, TAD boundary strength, and promoter/enhancer distance from TAD boundaries, to elucidate the role of genome structure in regulating gene expression. The applicability of our model is limited only to the availability of any chromosome conformation capture experiment data. As long as contact map is available, our model can be used to compare the relative changes in transcription in different chromatin organizations. We envision that our model will be particularly helpful in predicting the changes in E-P interactions and transcription due to nuclear deformation arising under various biological scenarios and in understanding the role of chromatin organizational changes during the cell development cycle and cell fate decisions.

Some single-cell micro-C assay experiments contradict the fact that E-P interactions remain intact due to CTCF loss^57^. It is possible that the correlation between specific E-P interactions and CTCF loss is missed due to the fixed-cell assay approach in the whole genome^44,61^. Recent high-resolution micro-C maps of fibroblast growth factor loci reveal miscommunication of enhancer-promoters due to loss of the CTCF array on the TAD boundaries^62^. Furthermore, Yokoshi et al. have reported that TAD disruption affects long-range E-P interaction in Drosophila by using quantitative live-imaging techniques^63^. These growing studies point in the direction of the importance of genome structure in regulating gene expression.

We briefly summarize the underlying assumptions of our model, although crucial to the model’s architecture. Firstly, our model considers chromatin dynamics within nucleoplasm which exhibits viscosity ranging from 0.03 P to 0.1 P^21,22,36^, which may depend on several factors such as the state of cell and cell type. To understand how viscosity may affect our final results, we simulated the specified genomic region for a range of viscosity values and were able to reliably reproduce E-P cluster size distribution for all viscosity values (Supplementary Fig. S2). It should be noted that viscosity affects the relaxation dynamics of the chromatin and short-term behavior may still depend on the viscosity values. Furthermore, each bead corresponds to a 10 kbp genomic region in our model, aligning with the resolution of experimental cHiC contact maps. However, it is important to acknowledge a resolution discrepancy between our model and the physical distribution of enhancers and promoters, which often span a smaller genomic locus with an average value of 50–1500 bps^1^. Despite this disparity, we can overlook this limitation, as for this particular genomic region, each enhancer falls into separate beads of the polymer model. An avenue for model improvement lies in increasing the polymer model’s resolution to better align with these finer genomic details, which again depend upon the resolution of the chromosome conformation capture experiment. In addition to that, in our kinetic model, we have assumed gene expression is proportional to the cluster of enhancers and transcription factors surrounding the promoter (see Section “Kinetic model”). This assumption about our model is based on a growing number of experimental studies in recent years. For example, Wang *et al*. have reported that YY1 activates gene expression of the FOXM1 gene by connecting enhancers and other coactivators with the FOXM1 promoter^64^. Furthermore, Lee et al. have elucidated that CTCF helps in transcription by forming clusters of RNA polymerase II, BRD4, and MED1 at the promoter and also helps in the looping of promoters and super-enhancers by forming transcriptional condensates^62^. Frazer et al. have documented that transcription factors facilitate the development of transcriptional condensates in living Candida albicans cells, as detected at the level of individual DNA molecules, and play a role in controlling cell fate determination^65^. Xiao et al. have also demonstrated that enhancers and general transcription factors form condensates at promoter sites to start the transcription in their GTF condensate model^44^ and could show hypersensitive transcriptional changes due to structural perturbations. We have also assumed uniform transcription factor binding rates for both promoters and enhancers due to the absence of detailed data and for the sake of model simplifications. Transcription factor (TF) binding is influenced by the specific binding sites present in promoters and enhancers^66–68^. Given that the *sox9* is located within a 10 kb genomic region and *kcnj2* within a distinct genomic region of similar resolution, we assume, for the purpose of model simplification, that TFs contribute similarly to the regulation of both genes. However, to enhance the quantitative accuracy and granularity of the model, it can be refined to account for gene-specific TF contributions derived from independent experimental data. While TF’s binding may indeed vary across different enhancers and promoters, it is also true that there are often common motifs or binding sequences that certain transcription factors recognize. In such cases, assuming equal binding and unbinding rates is a reasonable approximation when modeling the system at a coarse-grained level.

In summary, we have developed a computational method to predict the gene expression corresponding to the cHiC map. Through the utilization of this modeling technique, the complex regulatory network’s dynamic interactions are effectively captured. This not only contributes to the advancement of our fundamental comprehension of genome architecture but also offers significant insights into potential therapeutic interventions that aim to address abnormal gene regulation in pathological circumstances.

## Methods

We have developed a multistep computational framework to predict the relative gene expression level corresponding to the organization of chromatin characterized by any given chromosome conformation capture (3C) or 3C derived experiments (5C, HiC, cHiC etc.) contact map. The framework utilized a combination of cHiC contact maps and the genomic positions of enhancers and promoters as inputs. The first step of the approach is to determine three-dimensional chromatin conformations of the genomic region of interest through molecular dynamics simulation of the polymer model of chromatin.

### Polymer simulation

We employed molecular dynamics simulations to generate ensembles of three-dimensional chromatin conformations of the 6 Mb sox9-kcnj2 locus of chr11 in wildtype (WT) and deleted CTCF (DELC) cells of the mouse limb bud cells. A bead-spring polymer, which describes the polymer as beads connected by springs, was used to model the chromatin segment^69^. Our polymer consists of 587 beads in the WT cell and 586 beads in the DELC cell, each bead representing a 10 kb genomic region. This study adopts a methodological approach that extends upon the foundational framework for chromatin simulations established by Lappala et al.^70–72^.

We utilize a detailed particle-interaction potential used in polymer simulations. Polymer beads interact through the following interatomic potential with three components:

1 $$U={U}_{{FENE}}+{U}_{{LJ}}+{U}_{{harmonic}}$$

The consecutive beads interact with finite extensible nonlinear elastic (FENE) potential^69^

2 $${U}_{{FENE}}=-0.5K{R}_{0}^{2}\mathrm{ln}\left[1-{\left(\frac{r}{{R}_{0}}\right)}^{2}\right]+4\epsilon \left[{\left(\frac{\sigma }{r}\right)}^{12}-{\left(\frac{\sigma }{r}\right)}^{6}\right]+\epsilon$$

with length constant equal (${R}_{0}$) to 1.6*σ* and strength *K* of $\tfrac{30{k}_{B}T}{{\sigma }^{2}}$. To account for the excluded volume potential between any two non-bonded beads, the lennard-jones (LJ) potential^69^

3 $${U}_{LJ}=4\epsilon \left[{\left(\frac{\sigma }{r}\right)}^{12}-{\left(\frac{\sigma }{r}\right)}^{6}\right],r\, < \,{r}_{c}$$

is used with *σ* is the length scale unit, *ϵ* = *k*_*B*_*T* is the energy scale unit, and ${r}_{c}$ is the interaction range cutoff (1.12*σ*). The non-bonded beads are connected with harmonic constraint ${U}_{{Harmonic}}={k}{(r-{r}_{0})}^{2}$^73^, where the value of k depends on the contact probability (See Supplementary Fig. S1, Supplementary Section S1). The detailed optimization of parameters of harmonic constraints is discussed in the next subsection. The bead spring polymer was simulated using the LAMMPS simulation package^74^. The equation of motion is integrated by using the Verlet algorithm, while temperature is maintained at a constant value using a Langevin thermostat with a temperature of 1.0 (in reduced units) (see Supplementary Sections S1 and S3). The friction coefficient (*ζ*) which relates to the viscosity of the solvent (*η*) as $\zeta =6\pi \eta R$, where *R* represents the radius of the particle, was set to be 1.0^21,35,75^. As a range of viscosity values for nucleoplasm have been reported, we repeat our simulations for a range of friction coefficient values from 0.5 to 4 in reduced units (see Supplementary Fig. S2, Supplementary Section S1).

We started our simulation with a self-avoiding random walk and equilibrated the system in the NVT ensemble for 10^7^ timesteps. Following the equilibrium, we saved the trajectories at equal intervals to get 2 ×10^4^ distinct configurations of the considered chromatin region from each simulation. To enhance the sampling of configurations, we repeat the simulations with 200 different initial conditions and obtain a total of 4 ×10^6^ distinct configurations. These configurations were further used to compute the contact map and other analyses (see Supplementary Section S2). To validate our polymer modeling approach, we compared the E-P contact probabilities derived from both constrained and unconstrained polymer models with the experimental E-P interaction data (see Supplementary Section S4). We observed that the Pearson correlation between the simulated contact probabilities and the experimental contact frequencies was found to be approximately 0.9 for WT cells and 0.87 for DELC cells in the constrained polymer model, validating our polymer modeling approach (Supplementary Fig. S3).

### Polymer simulation constraint parameters

The non-bonded beads of the polymer are connected by harmonic springs with a contact-probability-dependent spring constant k to enforce desired configurations. Such a constraint is given by:

4 $${U}_{{Harmonic}}=k{(r-{r}_{0})}^{2}$$

where ${r}_{0}$ is the equilibrium bond distance. The harmonic spring constant k is assumed to be dependent on the contact probability by a power-law given as $k={k}_{0}{c}_{{ij}}^{\alpha },$ where ${k}_{0}$ is proportionality constant and *c*_*ij*_ is the input contact probability between the i^th^ bead and the j^th^ bead^29,30^. Different power law exponents *α*, were tested to determine the optimum relation between contact probabilities and the force constant k. We also used a range of ${k}_{0}$ values to get the best estimate of the considered chromatin structure by calculating the Pearson correlation coefficient between the simulated contact map and the experimental contact map (see Supplementary Section S1, Supplementary Fig. S1a, b). Following a systematic optimization, the constraint parameters demonstrating the strongest correlation with the input cHiC value were identified as *k*_0_ = 0.05 and *α* = 2. These optimized parameters were subsequently employed for all simulations and analyses. To further validate the robustness of our polymer model and address potential concerns about overfitting, we tested its performance by introducing Gaussian noise to the input Hi-C map. We applied two sets of noise with standard deviations of 0.1 and 0.5, respectively. The resulting polymer configurations generated by our model were compared to the modified Hi-C maps, yielding high Pearson correlation coefficients of 0.95 and 0.92 (Supplementary Fig. S4). These findings confirm that our model maintains strong predictive accuracy and stability even when the input data is perturbed, underscoring its robustness and mitigating concerns about overfitting.

### Generation of contact map

We have 200 simulations with different initial conditions, from which we have generated 4 ×10^6^ distinct polymer configurations for each WT and DELC cell types. These configurations were used to calculate the contact map based on the spatial distance. A pair is assumed to be in contact if distance between them is less than 1.2*σ* (Fig. 1c) (see Supplementary Section S2). We calculated the Pearson correlation coefficient between the simulated contact map and the experimental contact map as a measure of the accuracy of the model^29,30,35^. A high Pearson correlation coefficient signifies a robust linear association, implying that the simulated contact map closely mimics the experimental one^23,25,28^.

### Dataset

To validate our model with experiments, we choose a 6 Mb genomic region encompassing the sox9-kcnj2 locus of chromosome 11 in E12.5 mouse limb bud cells. All the simulation parameters including the contact map, position of enhancer and nucleoplasm viscosity were used for this loci. We have chosen these specific genomic loci as structural and expression data for structural variants is publicly available. Experimental cHiC data in WT cells and DELC cells along with transcription changes resulting from the deletion of the CTCF binding domain at the boundary region of TADs were obtained from GEO accession numbers GSE78109 and GSE125294 for WT and DELC cells, respectively^76^. Enhancers were identified by using VISTA^38^ and enhancer ATLAS 2.0^37^ databases. We identified 44 enhancers for sox9, which are consistent with the enhancers reported by Despang et al. for sox9 in the considered genomic loci. Six additional enhancers (mm628, mm629, mm630, mm631, mm632, and mm2181) were identified for the kcnj2 gene from these databases. For further validation of the model, we employed the exact framework on another 6MB genomic loci consisting pax3 gene of chromosome 1 in E11.5 mESC cell line and the experimental cHiC data for mutant cell lines (DELB and DELBS) of pax3 genomic loci were obtained from GEO accession number GSE92291^47^.

### Kinetic model

To investigate how enhancer-promoter (E-P) interactions and the assembly of transcription factors (TFs) at promoters regulate gene expression levels in WT and DELC *sox9* and *kcnj2* genes, we developed a stochastic simulation model (kinetic model) of gene expression. This model includes two promoters corresponding to the sox9 and kcnj2 genes. In this model, enhancers and TFs can bind to these promoters and initiate gene transcription. Enhancers bind and unbind to sox9 and kcnj2 promoters at rates ${b}_{{ij}}$ and ${u}_{{ij}}$, respectively, where i denotes individual enhancers and j denotes the promoters (j = 1 for sox9 and j = 2 for kcnj2). We derived these rates from the dwell time distribution of E-P interactions using the trajectories generated by our polymer simulation model (see Supplementary Section S5, Supplementary Figs. S5-S6). In the model, transcription factors bind and unbind to the promoters at rates r and g, respectively, which we set to 0.02 and 0.25^44^.

The average condensate formed by the combination of TFs and enhancers at the promoter site determines the gene expression level^44,45,77^. TFs and enhancers both have distinct contributions to the condensate size^66,67^. Therefore, in line with previous studies^78,79^, we scaled the enhancer contribution by a scalar factor w. Since enhancers can bind to 2 to 3 pre-condensates in initiating transcription, this weight factor accounts for the effect of enhancer size and its activity in promoting condensate formation^79,80^. For this study, the value of ‘w' is considered to be 2. This simplification allows us to capture the essential role of enhancers in promoting condensate formation and gene expression while acknowledging that more complex regulatory mechanisms may be at play. Despite this approximation and the limitations of the current dataset, our model provides a framework for integrating enhancer and TF data to predict gene expression levels.

Thus, the condensate size at the promoter of the j^th^ gene

5 $${C_j}=w{{n}}^{E}_j+{n}^{{TF}}_j$$

Here, ${n}^{E}_j$ and $n^{TF}_j$ are the number of enhancers and TFs attached to the promoter j, respectively, and w is the scaling factor. The time evolution of condensate size (Eq. (5)) for each of the promoters can be expressed by the following equation:

6 $$\frac{{dC}_{j}}{dt}=w\left(\sum _{i=1}^{N}{b}_{ij}{\delta }_{{m}_{i}}^{0}-\sum _{i=1}^{N}{u}_{ij}\times {\delta }_{{m}_{i}}^{j}\right)+(r-g\times {\delta }_{{m}_{i}}^{j})$$

Here, ${\delta }_{{m}_{i}}^{j}$ is the Kroneker delta, *m*_*i*_ = {0,1,2} represents the state of enhancer i {0 indicates free in the medium, 1 bound to promoter sox9, or 2 bound to promoter kcnj2} and N represents the total number of enhancers. In Eq. (6), the first term represents the increase in condensate size resulting from enhancer binding and second term accounts for decrease in size due to enhancer unbinding, and the last two terms represent the changes in condensate size due to TF binding and unbinding. We simulate the dynamics captured by Eq. (6) using Gillepsile’s algorithm^81,82^, and compute the condensate size as a function of time, generating the simulation trajectories, which we used to compute average condensate size as a function of time. Then, by assuming a direct proportional relationship between condensate size and gene expression level, this model enables us to measure the expression level of a gene. The code is written in MATLAB R2022A for the kinetic model simulation.

### Kinetic model parameters

All enhancers within the selected loci were identified, and the dwell time distributions were computed for each E-P pair using polymer simulation trajectories (Supplementary Fig. S5). Binding and unbinding rates were computed by fitting exponential curves to the dwell time distributions^83^, assuming E-P binding and unbinding as a Markovian process (see Supplementary Section S5). We also calculated the cluster-size dependent and enhancer-promoter pair independent rates from the cluster size distribution data (see Supplementary Fig. S6 and Supplementary Section S5).

## Supplementary information

supplementary Material

## Data Availability

This study utilizes publicly available data under the GEO accession numbers GSE78109 and GSE125294 for sox9-kcnj2 genomic loci and GSE92291 for pax3 genomic loci. Additional data can be found in the supplementary material.

## Code availability

All the codes and input scripts are available in the github link: https://github.com/CNRG-IITBBS/Bead-spring-polymer.
