# Supplementary material for: Predicting gene expression changes from chromatin structure modification
Source: NPJ Syst Biol Appl. 2025 Apr 15;11:34. doi: 10.1038/s41540-025-00510-4 (PMC12000410; doi:10.1038/s41540-025-00510-4)
Supplement: Supplementary file 1 — supplementary Material [file 41540_2025_510_MOESM1_ESM.docx]

**Supplementary Information**

**Predicting Gene Expression Changes from Chromatin Structure Modification**

Swayamshree Senapati^1^, Inayat Ullah Irshad^2^, Ajeet K. Sharma^2,3^ and Hemant Kumar^1,*^[^✉️^](mailto:hemant@iitbbs.ac.in)

^1^School of Basic Sciences, Indian Institute of Technology Bhubaneswar, Argul, Odisha 752050, India

, ^2^Department of Physics, Indian Institute of Technology Jammu, Jammu, 181221 India

^3^Department of Biosciences and Bioengineering, Indian Institute of Technology Jammu, Jammu, 181221 India

** Corresponding Author: Hemant Kumar, Email: hemant@iitbbs.ac.in*

1. **Polymer Simulation Constraint Parameters :**

To enforce chromatin conformation on the polymer model, we apply harmonic constraints between different beads such that magnitude of the spring force constant is proportional to contact probabilities from cHiC map. To prevent overfitting, these harmonic constraints are applied only to bead pairs with contact probabilities exceeding 0.5, which accounts for 40% of the contacts. Different power law scalings were tested to determine the optimum relation between contact probabilities and force constant k. Our calculations suggest that k ∝ c^2^_ij_ generates the conformations which exhibit the best correlation with cHiC (Figure S1a). The proportionality constant ($k_{0}$) between force constant (k) and contact probability (c_ij_) was varied for different power law scalings and $k_{0}$=0.05 generates the ensemble with the best Pearson correlation coefficient between experimental and simulated contact maps (Figure S1b). Final constraints are defined as $k=k_{0}c_{ij}^{\alpha}$ , where $k_{0}$ is 0.05 and $\alpha$ is 2. This constraint relation was used for all subsequent simulations and analyses.


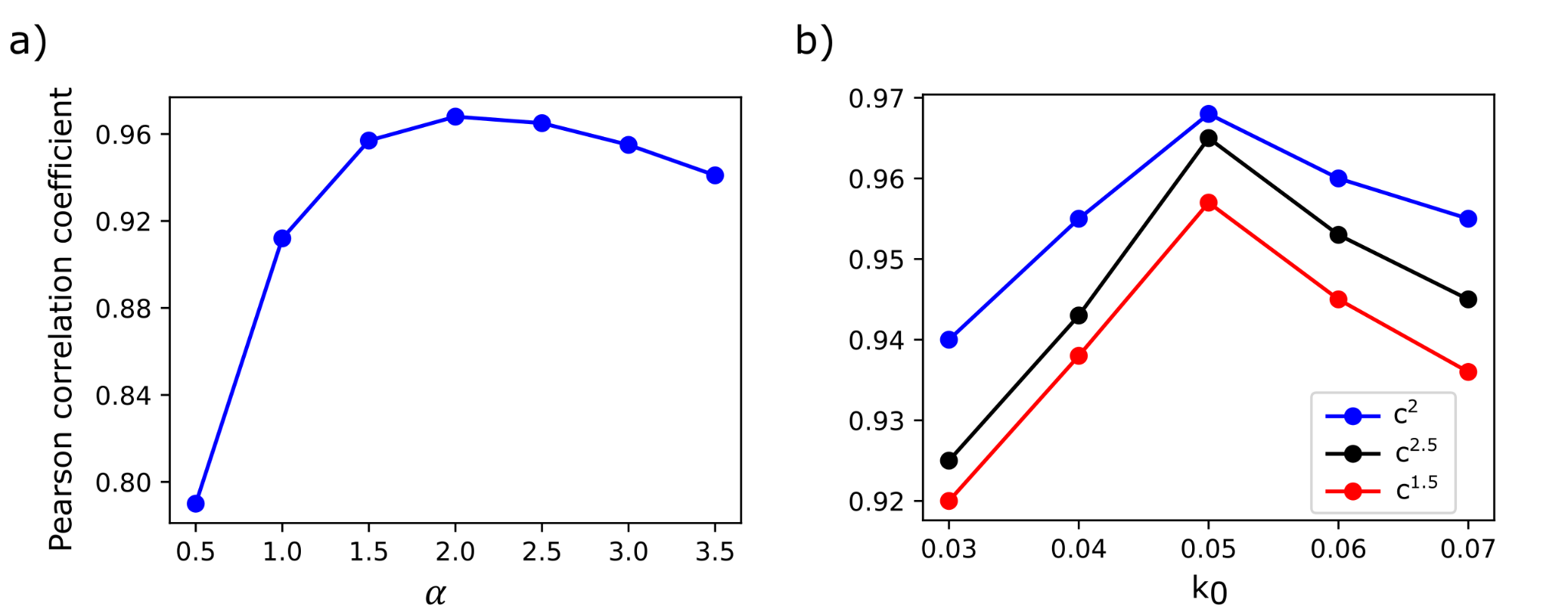


***Figure S1:*** *Harmonic constraint parameters* ***a)*** *Parameterization of* $C_{ij}^{\alpha}$*. The X-axis shows the variation of* $\alpha$*and the Y-axis shows corresponding Pearson correlation coefficient for proportionality constant* $k_{0}$*0.05;* ***b)*** *Parameterization of* $k_{0}$*. The X-axis shows the variation of* $k_{0}$*and the Y-axis shows corresponding Pearson correlation coefficient for* $C_{ij}^{1.5} (Red)$*,* $C_{ij}^{2}$ *(Blue),* $C_{ij}^{2.5} (Black)$*.*

We used the LAMMPS package (release: 8 April 2021) to model the bead spring polymer [[1]](https://www.zotero.org/google-docs/?Vmn9Dt). The equation of motion is integrated using the Verlet algorithm, and temperature is kept constant using Langevin thermostat (T = 1.0) [[2]](https://www.zotero.org/google-docs/?QRcVIe). The nucleoplasm was modeled as a viscous fluid with friction coefficient of a particle 𝜁 = 1.0 in reduced units surrounding the polymer. To ensure that our key results are not affected by the choice of parameters, we have reproduced key results with five different values of nucleoplasm viscosity (Figure S2) [[3], [4]](https://www.zotero.org/google-docs/?gr4zxL).

***Figure S2:*** *The probability distribution of enhancer cluster size for different friction coefficient values. From 0.5 to 4 in reduced units. The maximum number of enhancers around the promoter is at three for each of the friction coefficient values.*
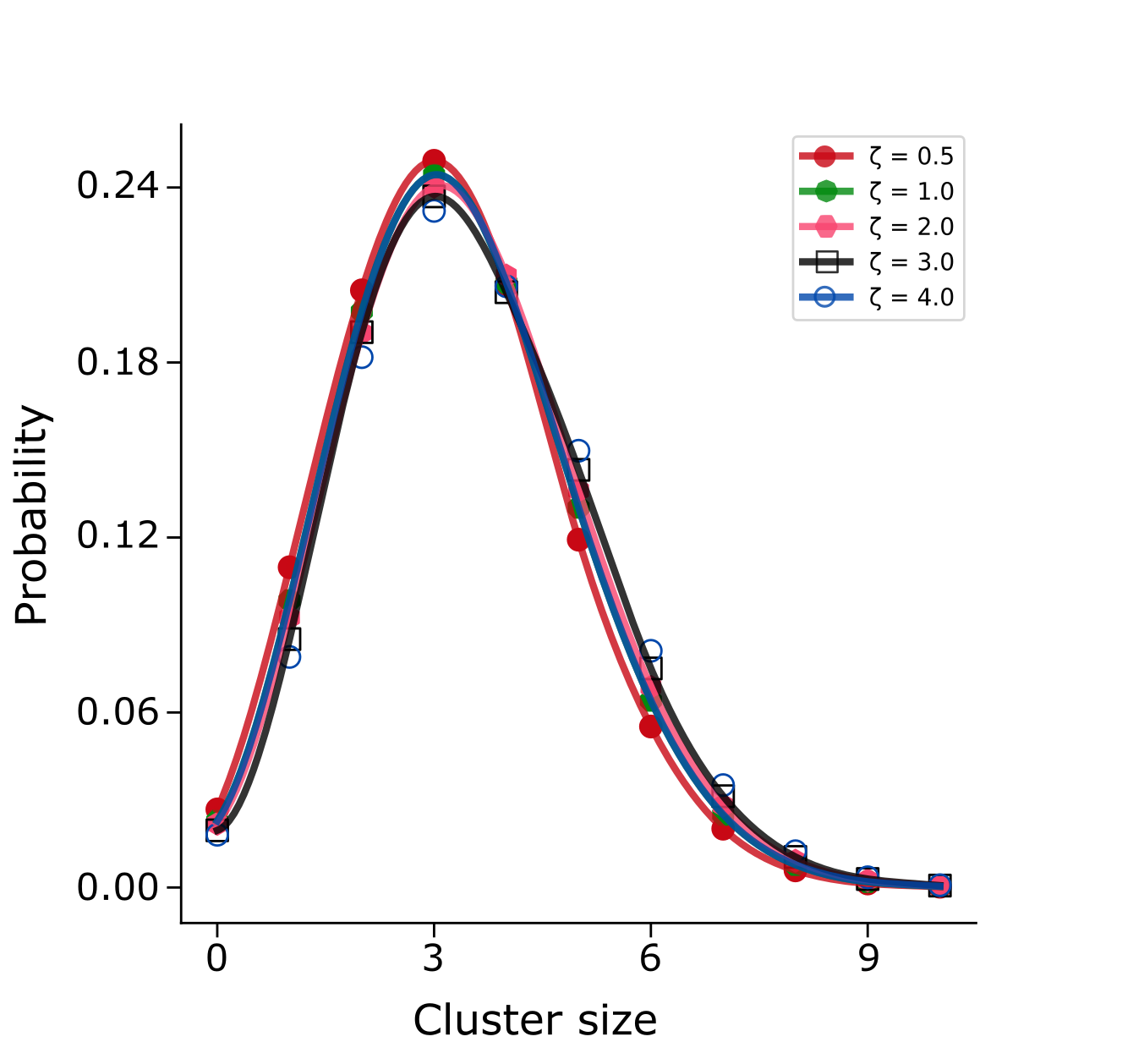


1. **Generation of Contact Map:**

We conducted 200 separate simulations for various initial conditions, resulting in the generation of $4 \times{10}^{6}$ unique configurations for each WT and DELC cell type. These configurations were used to determine the contact map for the spatial distance between them that is less than 1.2$\sigma$ [[5], [6]](https://www.zotero.org/google-docs/?ZtVHjh).

1. **Conversion of reduced units to physical units:**

For sake of simplicity, we have performed the polymer simulations in reduced units. We set the diameter of each bead in the polymer simulation to $\sigma$ and take it as unit length, and the mass of each bead to be unit mass. The energy scale unit is considered as $\epsilon=k_{\beta}T$. These reduced units can be mapped to physical units by selecting appropriate values from available experiments. For instance, the physical diameter of the bead $\sigma$ is estimated by following the same approach as Chiariello *et al.* and is estimated to be 75 nm, and if we take viscosity to be 0.1P and temperature to be 300K, then the time unit turns out to be 0.1 sec [[2]](https://www.zotero.org/google-docs/?sFtu5o).

1. **Validation of polymer model approach:**

To further assess the effectiveness of harmonic constraints in polymer model simulations, we performed simulations of an unconstrained polymer chain consisting of 587 beads. Starting from a self-avoiding walk (SAW) configuration, we used the FENE potential for consecutive beads and the Lennard-Jones (LJ) potential for non-bonded beads under periodic boundary conditions. This approach generated a three-dimensional conformation of the polymer chain without imposing any additional constraints between non-bonded beads [[7], [8], [9]](https://www.zotero.org/google-docs/?T35tFS). The resulting contact probability (pc​) followed a power law with genomic distance (s) as pc ~ $s^{-1.3}$. We compared the enhancer-promoter (E-P) contact probabilities obtained from both constrained polymer simulations and unconstrained polymer chain models with experimental E-P contact frequency data reported by Despang *et al.* [[10]](https://www.zotero.org/google-docs/?RJZYvv). The Pearson correlation between the simulated contact probabilities and the experimental contact frequencies was found to be approximately 0.9 for wildtype cells and 0.87 for CTCF-deleted cells in the constrained polymer model. In contrast, the corresponding correlations for the unconstrained ideal polymer chain were 0.29 and 0.2 for wildtype and CTCF-deleted cells, respectively (Figure S3). This suggests that harmonic constraints are effective in achieving the enabling of non-trivial contacts.

***Figure S3:*** *The bar plot shows the Pearson correlation coefficient between contact probabilities of enhancers and promoters between simulated and experimental data for constrained and unconstrained polymer chains for WT (blue bars) and DELC cells (green bars).*
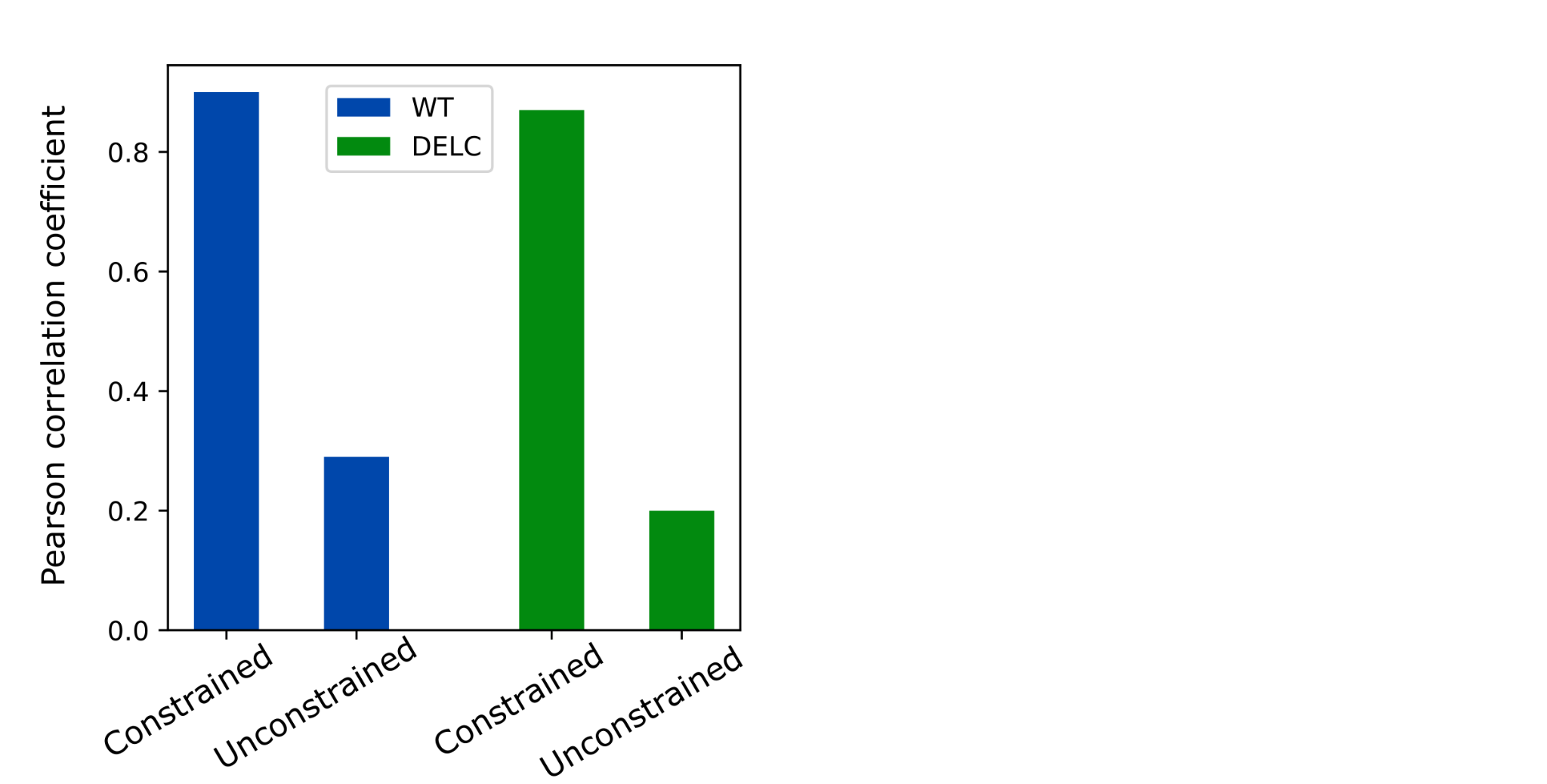


To validate the robustness of our polymer model and address overfitting concerns, we introduced Gaussian noise (σ = 0.1 and 0.5) to the input WT sox9-kcnj2 loci Hi-C map. The model maintained high predictive accuracy with Pearson correlation coefficients of 0.95 and 0.92, confirming its stability (Figure S4).


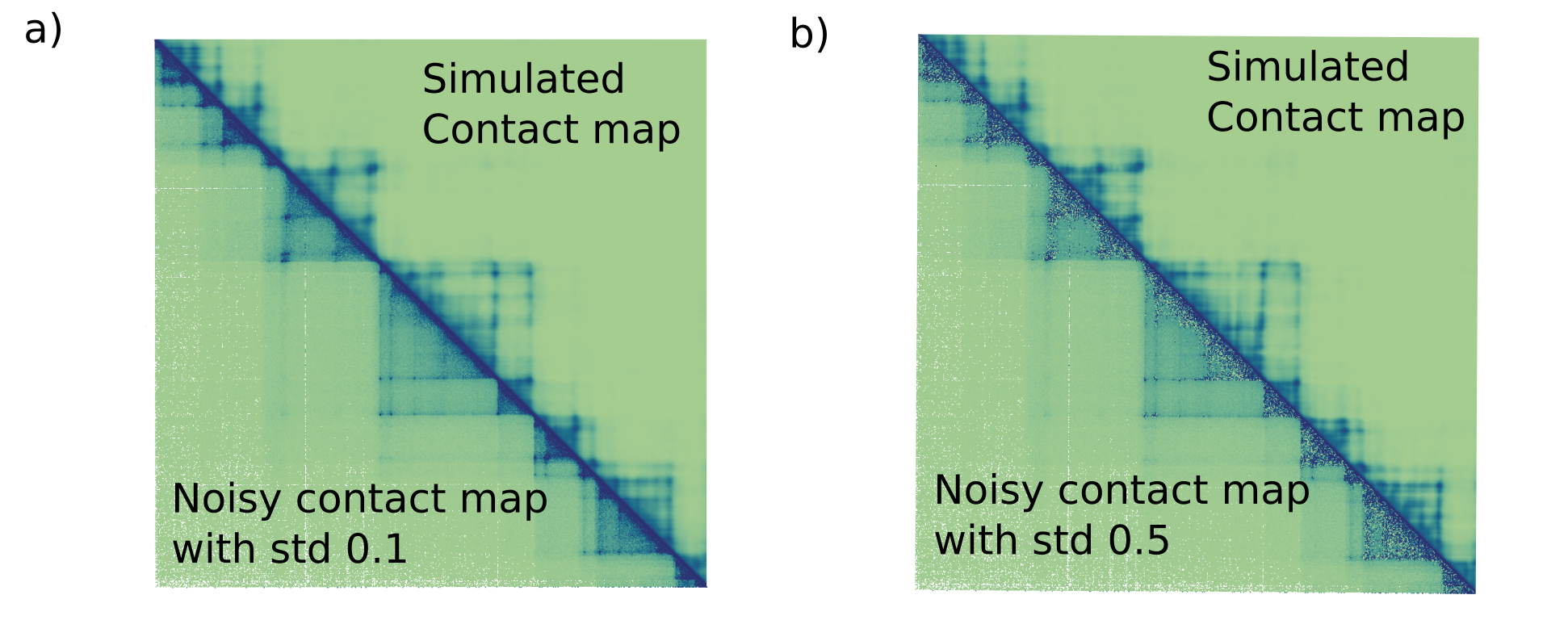


***Figure S4:*** *a) The contact map of input data of WT sox9-kcnj2 loci with added gaussian noise with standard deviation a) 0.1 (R=0.95), b) 0.5 (R=0.92) and the simulated contact map.*

1. **Analysis from the three-dimensional polymer structure and parameterization of Kinetic Model:**

We analyzed all chromating configurations generated from the polymer simulation trajectory to quantify the kinetics of E-P interactions. First, we computed binding and unbinding dwell time distributions for each enhancer-promoter pair from all the 200 distinct simulation trajectories. The binding dwell time refers to the duration for which an enhancer remains bound to a promoter, while the unbinding dwell time refers to the duration for which it remains unbound [[11]](https://www.zotero.org/google-docs/?8b1SUu). We fitted the dwell time distribution curve for each enhancer-promoter pair to a double exponential curve: $y=a{exp}^{-b_{ij}\tau}+$ $b{exp}^{-{b'}_{ij}\tau}$. As the slow process will be rate limiting, we consider the slower rates as binding rate (b_ij_) and unbinding rate (u_ij_) of enhancers with the promoter (Figure S5).


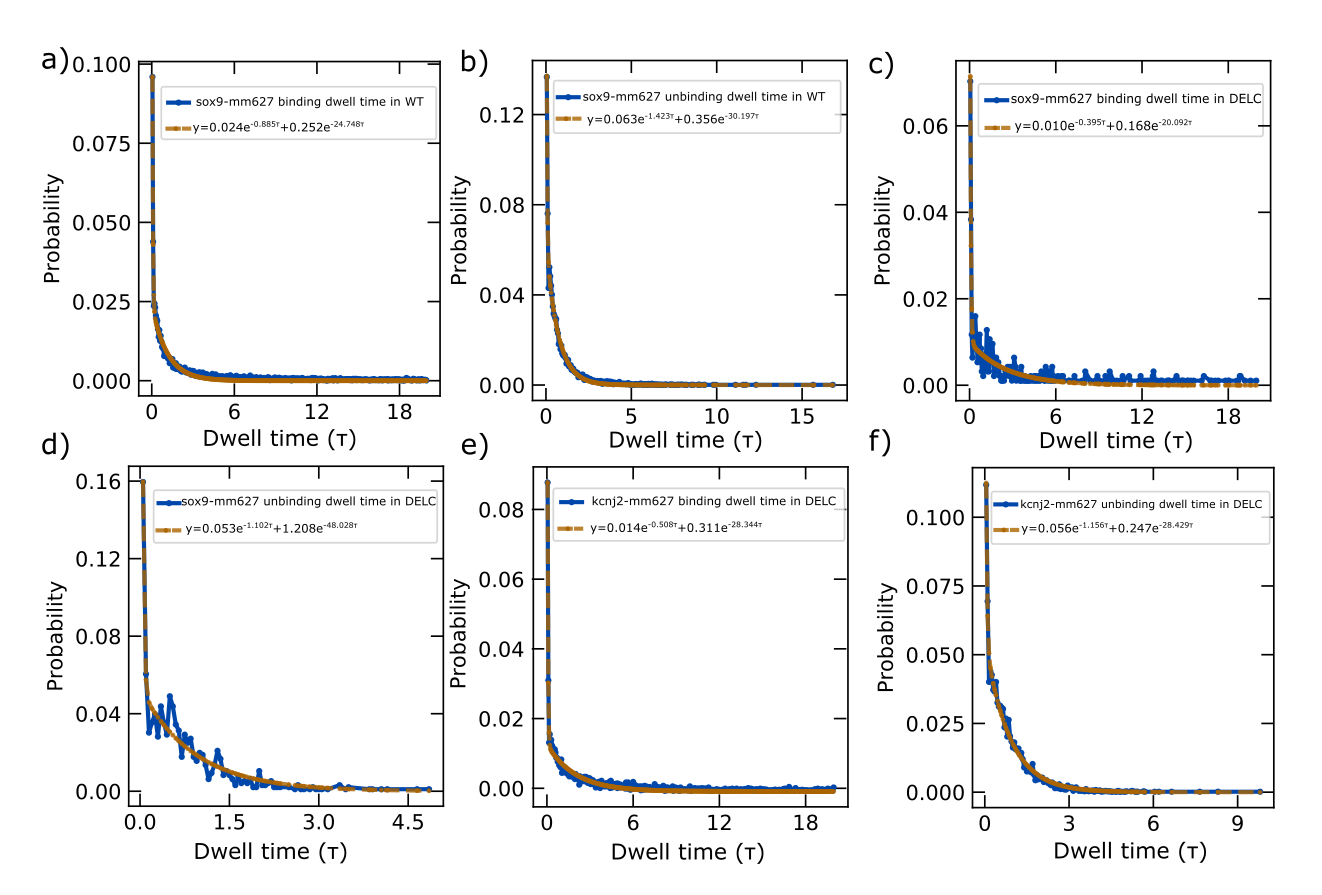


***Figure S5:*** *Exponential fitting of probability distribution of dwell time (in Lennard Jones or LJ units) of enhancer-promoter pairs* ***a)*** *binding of mm627 enhancer with sox9 promoter in WT cell;* ***b)*** *unbinding of m627 enhancer with sox9 promoter in WT cell;* ***c)*** *binding of mm627 enhancer with sox9 promoter in DELC cell;* ***d)*** *unbinding of m627 enhancer with sox9 promoter in DELC cell;* ***e)*** *binding of mm627 enhancer with kcnj2 promoter in DELC cell;* ***f)*** *unbinding of kcnj2 enhancer with sox9 promoter in DELC cell. The solid line represents the simulation dwell time data and the dashed line is the fitted curve.*

As the binding/unbinding rates of an E-P pair is likely to depend on the number of enhancers already bound to the promoter, we also calculated the cluster size-dependent rates. To determine the cluster size-dependent binding rate, we computed the dwell times to make a transition from one cluster size to the next larger size. Similar to individual E-P pairs, we fitted a double exponential to the dwell time distributions for each cluster size and found out the binding/unbinding rates for each cluster size. As shown in figure S6, both binding and unbinding rates exhibit a linear dependence on the size of the cluster (n). The slope of these curves was used to compute the rates for specific E-P pairs for both WT and DELC cells.

***
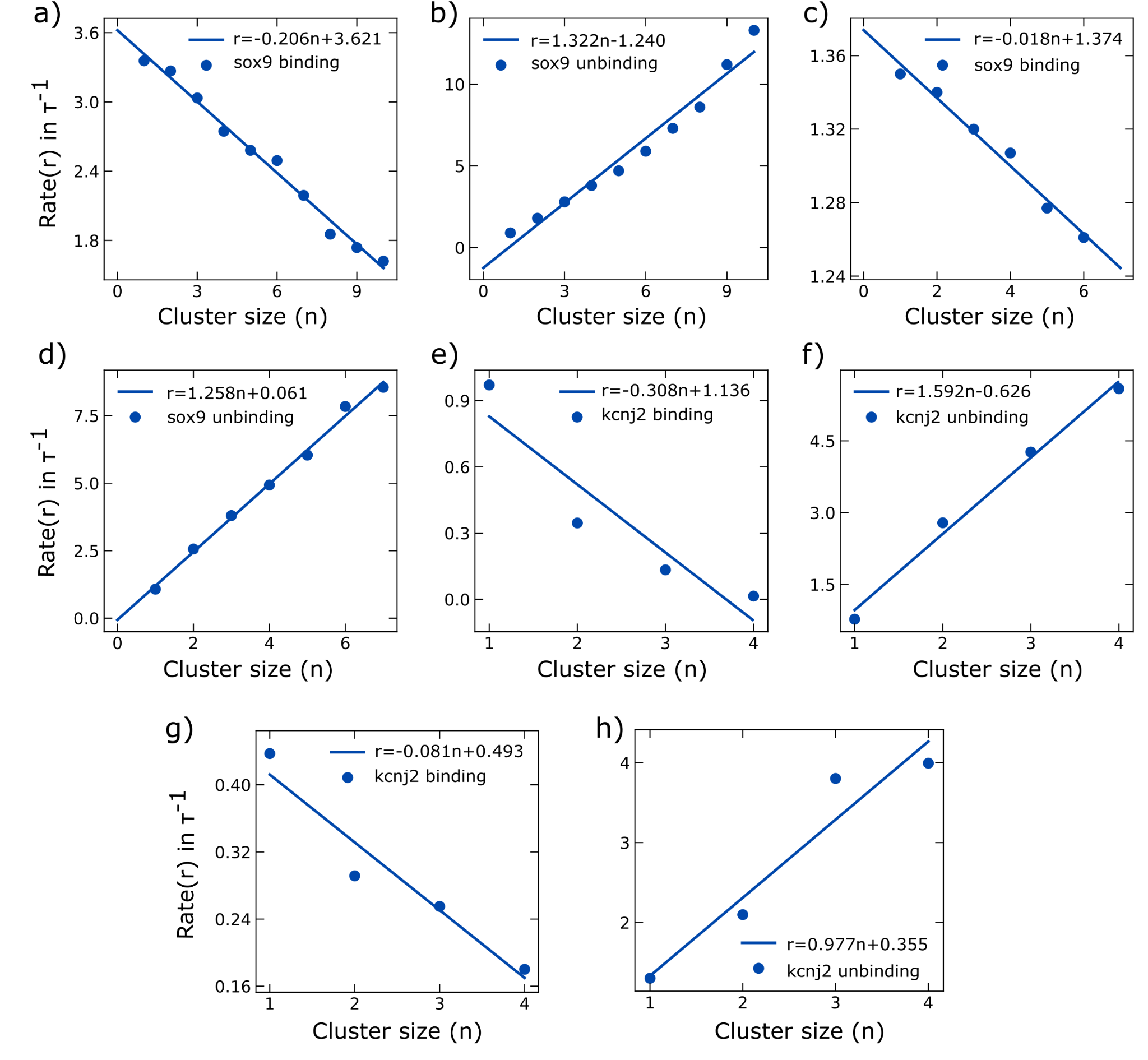
Figure S6:*** *Cluster-dependent binding and unbinding rates of enhancers with the promoters.* ***a)*** *Binding rates of enhancers for different cluster sizes (n) for sox9 in WT cell;* ***b)*** *unbinding rates of enhancers for different cluster sizes for sox9 in WT cell;* ***c)*** *binding rates of enhancers for different cluster sizes for sox9 in DELC cell;* ***d)*** *unbinding rates of enhancers for different cluster sizes for sox9 in DELC cell;* ***e)*** *binding rates of enhancers for different cluster sizes for kcnj2 in DELC cell;* ***f)*** *unbinding rates of enhancers for different cluster sizes for kcnj2 in DELC cell;*  ***g)*** *and* ***h)*** *binding and unbinding rates of enhancers for different cluster sizes of kcnj2 promoter in DELC cell (considering only specific enhancers), respectively. The solid line shows the fitted curve.*

**References:**

[[1] S. Plimpton, “Fast Parallel Algorithms for Short-Range Molecular Dynamics,” *J. Comput. Phys.*, vol. 117, no. 1, pp. 1–19, Mar. 1995, doi: 10.1006/jcph.1995.1039.](https://www.zotero.org/google-docs/?THHV1S)

[[2] A. M. Chiariello, C. Annunziatella, S. Bianco, A. Esposito, and M. Nicodemi, “Polymer physics of chromosome large-scale 3D organisation,” *Sci. Rep.*, vol. 6, no. 1, Art. no. 1, Jul. 2016, doi: 10.1038/srep29775.](https://www.zotero.org/google-docs/?THHV1S)

[[3] M. Baum, F. Erdel, M. Wachsmuth, and K. Rippe, “Retrieving the intracellular topology from multi-scale protein mobility mapping in living cells,” *Nat. Commun.*, vol. 5, no. 1, Art. no. 1, Jul. 2014, doi: 10.1038/ncomms5494.](https://www.zotero.org/google-docs/?THHV1S)

[[4] C. A. Brackey, D. Marenduzzo, and N. Gilbert, “Mechanistic modeling of chromatin folding to understand function,” *Nat. Methods*, vol. 17, no. 8, Art. no. 8, Aug. 2020, doi: 10.1038/s41592-020-0852-6.](https://www.zotero.org/google-docs/?THHV1S)

[[5] G. Fudenberg, M. Imakaev, C. Lu, A. Goloborodko, N. Abdennur, and L. A. Mirny, “Formation of Chromosomal Domains by Loop Extrusion,” *Cell Rep.*, vol. 15, no. 9, pp. 2038–2049, May 2016, doi: 10.1016/j.celrep.2016.04.085.](https://www.zotero.org/google-docs/?THHV1S)

[[6] M. Conte, L. Fiorillo, S. Bianco, A. M. Chiariello, A. Esposito, and M. Nicodemi, “Polymer physics indicates chromatin folding variability across single-cells results from state degeneracy in phase separation,” *Nat. Commun.*, vol. 11, no. 1, Art. no. 1, Jul. 2020, doi: 10.1038/s41467-020-17141-4.](https://www.zotero.org/google-docs/?THHV1S)

[[7] E. Lieberman-Aiden *et al.*, “Comprehensive Mapping of Long-Range Interactions Reveals Folding Principles of the Human Genome,” *Science*, vol. 326, no. 5950, pp. 289–293, 2009, doi: 10.1126/science.1181369.](https://www.zotero.org/google-docs/?THHV1S)

[[8] S. S. P. Rao *et al.*, “Cohesin Loss Eliminates All Loop Domains,” *Cell*, vol. 171, no. 2, pp. 305-320.e24, Oct. 2017, doi: 10.1016/j.cell.2017.09.026.](https://www.zotero.org/google-docs/?THHV1S)

[[9] B. Chan and M. Rubinstein, “Theory of chromatin organization maintained by active loop extrusion,” *Proc. Natl. Acad. Sci.*, vol. 120, no. 23, p. e2222078120, Jun. 2023, doi: 10.1073/pnas.2222078120.](https://www.zotero.org/google-docs/?THHV1S)

[[10] A. Despang *et al.*, “Functional dissection of the Sox9-Kcnj2 locus identifies nonessential and instructive roles of TAD architecture,” *Nat. Genet.*, vol. 51, no. 8, pp. 1263–1271, Aug. 2019, doi: 10.1038/s41588-019-0466-z.](https://www.zotero.org/google-docs/?THHV1S)

[[11] N. C. Lammers, Y. J. Kim, J. Zhao, and H. G. Garcia, “A matter of time: Using dynamics and theory to uncover mechanisms of transcriptional bursting,” *Curr. Opin. Cell Biol.*, vol. 67, pp. 147–157, Dec. 2020, doi: 10.1016/j.ceb.2020.08.001.](https://www.zotero.org/google-docs/?THHV1S)

###

###

###

### **Checklist for Computational Models in Gene Expression and Chromatin Structure**

#### **1. Title and Abstract**

- Does the title clearly indicate the study's focus on gene expression prediction through chromatin structure modeling?
- Is the abstract concise and includes the key elements such as the objective, methods (computational framework, polymer model), results (impact of CTCF boundary deletion), and conclusion?

#### **2. Introduction**

- Is the scientific context of the study clearly defined, explaining the importance of chromatin organization in gene regulation?
- Does the introduction provide an overview of the gap in knowledge that the study addresses (e.g., the relationship between chromatin structure and gene expression)?
- Is the rationale for using computational models (bead-spring polymer model and cHiC data) explicitly stated?

#### **3. Methods**

- **Computational Framework**:
  - Does the methodology clearly describe the computational model and its components (bead-spring polymer model, Markov-chain model, cHiC contact map)?
  - Is the use of contact maps (HiC, cHiC) explained in terms of how they inform chromatin conformation simulations?
  - Is the Markov-chain model and its parameters (binding/unbinding rates) detailed sufficiently for reproducibility?
- **Simulations and Analysis**:
  - Are the simulation parameters (e.g., polymer model configuration, kinetic model of gene expression) clearly outlined?
  - Is there an explanation of how the TAD boundary deletion affects the structure and enhancer-promoter interactions?
  - Are the criteria for identifying functional enhancers from the model results clearly described?
- **Data and Software**:
  - Are all data sources (cHiC) and software tools used (e.g., simulation software) clearly identified?
  - Is the version of the software or scripts used mentioned?

#### **4. Results**

- Are the results presented in a clear, systematic manner?
- Are key findings from the simulations (e.g., changes in enhancer-promoter interactions due to TAD boundary deletion) reported quantitatively?
- Are any figures, tables, or visual aids used to illustrate the predicted gene expression changes and chromatin structural modifications?

#### **5. Discussion**

- Are the findings interpreted in the context of existing research on chromatin organization and gene expression regulation?
- Are limitations of the computational framework (e.g., assumptions, resolution of HiC data) acknowledged?

#### **7. References**

- Are all cited studies (HiC, molecular dynamics, chromatin models, etc.) included and appropriately referenced?
- Are all references relevant and up-to-date?

#### **8. Code availability statement and Supplementary Information**

- Are any additional materials (e.g., code, datasets) provided for replication and transparency?
- Are detailed descriptions of computational methods or equations included?
